# Genetics of Cushing’s disease: from the lab to clinical practice

**DOI:** 10.1007/s11102-022-01253-9

**Published:** 2022-07-19

**Authors:** Marily Theodoropoulou, Martin Reincke

**Affiliations:** Medizinische Klinik und Poliklinik IV, LMU Klinikum, Ludwig-Maximilians-Universität München, Ziemssenstr. 5, 80336 Munich, Germany

**Keywords:** Cushing’s disease, Corticotroph tumor, Genetics, USP8

## Abstract

Cushing’s disease is a rare, but devastating condition, caused by corticotroph tumors. It rarely manifests as syndrome and very few isolated cases present with germline mutations. Instead, the vast majority of corticotroph tumors are sporadic monoclonal neoplasms. At present, the major recurrent somatic driver mutations are found in the *USP8* gene, which encodes for a deubiquitinase that rescues proteins regulating ACTH synthesis. Almost half of functional corticotroph tumors carry somatic *USP8* mutations that associate with a distinct transcriptomic and clinical profile. Other genes mutated in a small fraction of corticotroph tumors include the deubiquitinase encoding gene *USP48* and the glucocorticoid receptor expressing *NR3C1*. Recent reports on somatic *TP53* and *ATRX* mutations in corticotroph macroadenomas and carcinomas indicate that within specific patient subpopulations they are not as rare as assumed.

## Introduction

In the early 1930’s, “*basophil adenomas of the anterior lobe of the pituitary*” were established as the cause of Cushing’s disease [1]. Flash forward 50 years, advancements in molecular techniques established their monoclonality, but hypothesis based analyses failed to identify driver gene alterations (reviewed in [2]). The advent of next generation sequencing led to the discovery of the *USP8* mutational hotspot and prompted us to revisit genes that were previously studied but considered to be rarely mutated, such as *NR3C1* encoding for the glucocorticoid receptor and the tumor suppressor gene *TP53*. This short review summarizes our current knowledge on mutational events found in functional corticotroph tumors.

## Germline mutations

Cushing’s disease is very rare in genetic syndromes, such as, multiple endocrine neoplasia 1 (*MEN1* encoding for menin), MEN4 (*CDKN1B* encoding for the cell cycle inhibitor p27/Kip1) and Carney complex (*PRKAR1A* encoding for type 1 alpha regulatory subunit of the cAMP-dependent protein kinase A) (reviewed in [2, 3]). Similarly, it is very rarely found in patients with familial isolated pituitary adenomas (FIPA). Screening pediatric and adult patients with evidently sporadic corticotroph tumors revealed very few cases with germline mutations in *CDKN1B* or *AIP* (aryl hydrocarbon receptor-interacting protein; mutated in ~ 10% of FIPA).

DICER1 syndrome (inactivating mutations in the gene encoding for the type III endoribonuclease Dicer involved in small non-coding RNA processing) is a rare pediatric condition that predisposes to pleuropulmonary blastoma and other dysplasias. Cushing’s disease often manifests in the rare cases (< 1%) of DICER1 patients with unique pituitary blastoma [4].

Lynch syndrome predisposes to several cancers and is caused by germline mutations in DNA mismatch repair genes (e.g. *MSH2*, *MSH6* and *PMS2*). Although pituitary tumors are seldom, when present they are often invasive corticotroph tumors or carcinomas (reviewed in [3]).

Screening of 182 patients with corticotroph tumors (including 116 pediatric) identified germline missense variants in *CABLES1* (CDK5 and ABL1 enzyme substrate 1; mediates the antiproliferative action of glucocorticoids in corticotroph tumor cells) in 4 patients (2 pediatric) with macroadenomas (> 10 mm) and difficult to manage disease [5].

## Sporadic

Cushing’s disease occurs mainly in a sporadic setting. Somatic mutations in genes involved in the trophic hypothalamic signaling are rare (guanine nucleotide binding protein alpha stimulating, *GNAS*) or nonexistent (corticotrophin releasing hormone receptor 1, *CRHR1*; vasopressin V3 receptor, *V3R*). Regarding the negative glucocorticoid feedback, somatic mutations in the *NR3C1* (nuclear receptor subfamily 3 group C member 1) gene encoding for glucocorticoid receptor were initially considered as extremely rare, but recent sequencing efforts identified them in 0–10% of corticotroph tumors (reviewed in [2]). No distinguishing clinical parameters were attributed to *NR3C1* mutants, probably due to low numbers [6].

At present, the genetic basis of Cushing’s disease is recurrent somatic mutations in the ubiquitin specific protease 8 (*USP8*) gene found in almost half of functional corticotroph tumors, including pediatric and Nelson’s syndrome [7–10; reviewed in 2, 3]. All *USP8* mutations are somatic, with one germline mutation reported in a pediatric patient with severe Cushing’s disease (reviewed in [3]). The *USP8* mutational hotspot disrupts the 14-3-3 binding motif resulting in enhanced catalytic activity. In corticotroph tumor cells, these highly active USP8 mutants rescue epidermal growth factor receptor (EGFR) from ligand-induced ubiquitination and lysosomal degradation and potentiate its signaling resulting in enhanced ACTH synthesis [6]. USP8 mutant corticotroph tumors show female predominance and are smaller, less invasive, but more likely to recur after surgery. They also show increased somatostatin receptor 5 (SSTR5) expression, suggesting favorable response to the SSTR5 ligand pasireotide [10]. In vitro studies demonstrated a better response to the antisecretory action of pasireotide in human USP8 mutant corticotroph tumors [11]. USP8 wild type tumors, on the other hand, are found in both female and male patients and are usually macroadenomas and more likely to be aggressive [7–10].

A second mutational hotspot was found in ubiquitin specific protease 48 (*USP48*) in a small percentage (up to 23%) of *USP8* wild type tumors [12, 13]. Similar to *USP8*, *USP48* mutant tumors are more frequently found in female patients and are smaller.

An unexpected gene picked by whole exome sequencing is the *BRAF* proto-oncogene [12]. The *BRAF* V600E mutation was found in 16.5% of USP8 wild type corticotroph tumors in a Chinese patient cohort, where it correlated with higher midnight ACTH and serum cortisol, but in only 1/91 Caucasian cases (female patient with macroadenoma) and none in other Asiatic patient cohorts [12–14].

Activating somatic mutations in the *PIK3CA* gene that encodes for the p110α catalytic subunit and constitutively activate the PI3K/AKT survival pathway were found in 1 out of 6 invasive corticotroph tumors, which also harbored an oncogenic *HRAS* mutation, and in none of the 16 noninvasive corticotroph tumors [15]. A separate study reported *PIK3CA* mutations in 1 out of 6 corticotroph microadenomas [16].

Finally, there are increasing reports of somatic mutations in *TP53* and *ATXR* (alpha thalassemia/ mental retardation syndrome X-linked) in aggressive corticotroph tumors and corticotroph carcinomas. In selected populations of functional corticotroph tumors (i.e. USP8 wild type macroadenomas), *TP53* mutations were found in up to ~ 35% of cases [13, 17]. Similarly, *ATXR* variants were found in 7/25 aggressive corticotroph tumors and carcinomas that were ATXR immunonegative [18]. Few cases carried mutations in both *TP53* and *ATXR* or its interaction partner *DAXX* (death domain associated protein) [13, 18].

## Multi-omics

Epigenetic profiling revealed that corticotroph tumors group in distinct miRNome and methylome clusters [10, 19]. The methylome of functional corticotroph tumors shows few areas of hypomethylation that include the *POMC* promoter [10, 19]. Notably corticotroph tumors from Cushing’s disease patients cluster separately from silent corticotroph tumors in terms of methylome and transcriptome [10]. Enrichment analysis revealed high expression of cell cycle related genes in functional corticotroph tumors [10]. Interestingly, *USP8* wild type tumors show distinct transcriptomic profile with enrichment in epithelial-mesenchymal transition signatures, which may explain their more invasive nature [10].

## Conclusion

During the last five years, we have gained a vast amount of information on the mutational landscape of Cushing’s disease. The discovery of the driver USP8 mutational hotspot highlighted distinct clinical profiles of Cushing’s disease patients: those with *USP8* mutant tumors are predominantly female and less likely to go into remission after surgery, but have increased SSTR5 expression, which may indicate certain treatment strategies for the management of these tumors. In contrast, USP8 wild type tumors form a heterogeneous group that include tumors with no identified driver mutations, but also cases with *TP53* mutations and *ATXR* mutations, which may allude to a more aggressive tumor behavior that may require intense management and long-term follow up. Although not of relevance in the general context of Cushing’s disease, isolated reports of somatic *TP53* and *ATXR* mutations in aggressive corticotroph tumors and carcinomas, suggest that genetic *TP53* screening and/or ATXR immunohistochemistry in selected patient subpopulations may indicate those more likely to require increased surveillance. Finally, ongoing multi-omics analyses are expected to provide with additional tools for the improved diagnosis and monitoring of patients with Cushing’s disease.
